# Prevalence and determinants of microalbuminuria among diabetic patients in the United Arab Emirates

**DOI:** 10.1186/1471-2369-9-1

**Published:** 2008-01-29

**Authors:** Fatma Al-Maskari, Mohammed El-Sadig, Enyioma Obineche

**Affiliations:** 1 Department of Community Medicine, Faculty of Medicine & Health Sciences, Al-Ain City, United Arab Emirates University, PO Box: 17666, UAE; 2 Department of Internal Medicine, Faculty of Medicine & Health Sciences, Al-Ain City, United Arab Emirates University, PO Box: 17666, UAE

## Abstract

**Background:**

Microalbuminuria (MA) represents the earliest clinical evidence of diabetic nephropathy and is a predictor of increased cardiovascular morbidity and mortality. The aim of this study was to determine the prevalence of MA among diabetic patients in the Al-Ain district of the United Arab Emirates (UAE).

**Methods:**

The study was part of a general cross-sectional survey carried out to assess the prevalence of diabetes mellitus (DM) complications in Al-Ain district, UAE and was the first to assess the prevalence of MA among diabetic patients. A sample of 513 diabetic patients with a mean age of 53 years (SD: ± 13) was randomly selected during 2003/2004. All patients completed an interviewer-administered questionnaire and underwent medical assessment. First morning urine collections were obtained and were tested for clinical proteinuria using urine dipsticks and for MA using the single Micral-Test II strips.

**Results:**

MA was found in 61% (95% CI: 56.7–65.7) of the sample and the rate was significantly higher among males, positively related to body mass index (BMI), type 2 DM and presence of other DM complications such as diabetic retinopathy and neuropathy. Of the total sample population, 12.5% (95% CI: 8.1-14.1) had clinical proteinuria.

**Conclusion:**

The prevalence rate of MA  was considerably high ( 61%) among diabetic patients in the UAE. Therefore, regular screening for MA is recommended for all diabetic patients, as early treatment is critical for reducing cardiovascular risks and slowing the progression to late stages of diabetic nephropathy (overt proteinuria and end-stage renal disease).

## Background

Diabetes mellitus (DM) has long been recognized as a major public health problem with far reaching consequences, not only for its adverse health impact on individuals, but also for its economic burden on the health care system and the society at large [1]. The International Diabetes Federation (IDF) in 2005 confirmed that diabetes is one of the most common non-communicable diseases globally and constitutes the fourth or fifth leading cause of death in most developed countries as well as many developing and newly industrialized countries, such as the United Arab Emirates (UAE) [2]. The IDF in 2003 ranked the UAE's prevalence rate for type 2 DM and IGT as the second highest in the world (20% for DM and 26% for IGT) [2].

Diabetic nephropathy is characterized by proteinuria and is widely considered as the leading cause of end-stage renal disease (ESRD) which constitutes the major workload of dialysis centers worldwide. Indeed, the costs of dialysis and renal transplantation imposed by ESRD implicate a sizeable burden on health care resources [3] and seriously compromise both the quality of life and life expectancy [4-7]. It is well known that progression to established diabetic nephropathy occurs through several stages. MA, defined as urinary albumin excretion rate of 20–200 ug/min on a timed specimen without an alternative clinical explanation (such as urinary tract infection, heart failure or exercise in the past 48 hours) or urinary protein excretion rate of 30–300 ug/min, is a known predictor of future developments of overt diabetic nephropathy [8]. Because MA can be reversed and the future developments of overt diabetic nephropathy can be significantly reduced, screening for MA and the timely therapeutic intervention has become the standards of care worldwide.

Screening for MA can be performed using quantitative methods, including: i) measurement of albumin to creatinine ratios in a random urine sample; ii) a 24-hour collection with creatinine, allowing the simultaneous measurement of creatinine clearance; iii) timed (e.g. 4 hour) overnight urine collection for protein, or by using semi-quantitative reagent dipsticks specifically designed with detection limits suitable for identifying microalbuminuria, such as the Micral dipsticks [9].

According to the American Diabetes Association (ADA), when using the random collection technique, normal albumin excretion should be defined as <30 mcg/mg of creatinine; microalbuminuria 30 to 299 mcg/mg of creatinine, and macroalbuminuria is 300> mcg/mg of creatinine [10]. In the 24-hour collection technique, albumin excretion <30 mg per 24 hours is considered normal, 30 to 299 mg per 24 hours indicates microalbuminuria, and 300 mg or higher indicates macroalbuminuria [10]. When using the timed collection technique, normal albumin excretion is defined as <20 mcg/min, microalbuminuria is defined as 20 to 199 mcg/min, and macroalbuminuria as 200> mcg/min [10].

Screening for MA with Micral-test II strips is relatively cheap, fast and has an acceptable sensitivity of 97% with a specificity of 71% [9]. Several studies comparing Micral test II and laboratory methods of detecting albuminuria have concluded that the test could be used as a screening tool but, because of its low specificity, cannot be used as a diagnostic tool [11-13]. Micral test II is an optically read immunoassay, specifically designed for detection of MA and the use of these strips has been widely advocated [9].

Generally, screening for MA is relatively cheap and convenient procedure to detect MA among diabetic patients to reduce cardiovascular risks and the rate of progression of diabetes-related nephropathy. To our best knowledge our study constitutes the first effort in the UAE to estimate the prevalence of MA among patients with diabetes mellitus.

## Methods

### Overall design

The study was part of a general cross-sectional survey of DM patients carried out to assess and establish the prevalence of DM complications including MA among diabetic patients in Al-Ain District, UAE, using the single Micral-Test II strips.

### Subjects and setting

The sampling frame of this study included all nationals and non-nationals diabetic patients of all ages and both genders attending any primary health care center (PHCC) for any reason and diabetic clinics at hospitals for follow up in Al-Ain district of Abu Dhabi. Power and sample estimations were used to estimate a sample size with a standard error of 2%. Accordingly, a sample size of 625 patients was estimated. To achieve that all PHCC and diabetic clinics in the district were enlisted and enumerated and multistage random sampling was used to select 8 PHCC (a proportion of 25% out of 22 rural and urban PHCC) in addition to two diabetic clinics at the two main public hospitals in Al-Ain district. In the absence of a diabetes registry or a computerized database for patients in the district, systematic random sampling was used to select patients to be approached for participation in the study. Therefore, every third DM patient visiting the participant PHCC and diabetic clinics was approached. In total, 600 patients were approached by general practitioners and diabetologists, out of which 513 patients (86%) agreed to enroll. The study was approved by the Joint Ethics Committee of the Faculty of Medicine and Health Sciences of the UAE University and the Al-Ain Medical District. The data were collected between September 2003 and May 2004.

### Data collection

After receiving a prior informed consent (a written one from literate patients and a verbally informed one from illiterate patients), participant patients were interviewed by their treating doctors about DM type, duration, treatment profile, level of control, presence or absence of chronic DM complications. The diagnosis of diabetes was based on self reported physician diagnosis of diabetes. Participants who were not responding to oral hypoglycemic agents and needed insulin treatment within two years of diagnosis were classified as having type 1 diabetes. All other cases were classified as type 2 diabetes.

Blood pressure (BP) was measured early in the morning and prior to drawing of blood samples using a suitable mercury sphygmomanometer after a 10 minutes rest with the patient in the sitting position. BP was measured two times at 5 minutes interval. The first and the fifth *korotokoffs *sound were used to determine the systolic and diastolic blood pressure measurement, respectively. The second blood pressure measurement was used as the blood pressure for the individual. The WHO definition of hypertension was used in this study: systolic blood pressure 160 mmHg or more and/or a diastolic blood pressure 95 mmHg or more [14], or if the patient is on treatment with antihypertensive drugs. Height was measured without shoes, and weight was recorded while wearing indoor clothing. Body mass index (BMI) (weight in Kg, divided by height in meters squared) was calculated. The WHO (1995, 2002) classification for BMI was used to estimate the degree of obesity [15]. A standard 12-lead electrocardiogram (ECG) was recorded for all patients. Fasting blood samples were taken to assess lipid profile, blood sugar and glycated hemoglobin (HbA_1C_) levels. Total lipid profile (total cholesterol (TC), high density lipoprotein (HDL), TC/HDL ratio, low density lipoprotein (LDL) and triglycerides) was measured by a capillary tube whole blood method using the Cholesterol LDX lipid analyzer. Dyslipidaemia was taken to be present when the total cholesterol was >5.60 mmol/L and/or triglycerides >2.10 mmol/L, LDL >3.4 mmol/L, and/or HDL <0.91 mmol/L. Fasting blood glucose was measured by glucose oxidase method; Clinical Chemistry Analyzer. Glycated haemoglobin (HbA_1C_) was measured using the Bayer DCA 2000+ analyzer and a value of less than 7% was taken to indicate good glycemic control.

The diagnosis of diabetic nephropathy was made when MA and/or clinical proteinuria was detected and other causes of kidney disease or alternative explanations for proteinuria such as urinary tract infection (UTI), marked hypertension, heart failure and febrile illness were excluded. Patients who had undergone renal transplantation or were treated with dialysis with no other reason for renal failure were also considered to have diabetic nephropathy. Proteinuria was defined by the presence of protein in the urine using the conventional test strips (Redia, Boehringer-Mannheim, Mannheim, Germany). Two categories of proteinuria were identified: severe level; in those with heavy proteinuria (≥3+); and trace or intermediate level in those who had (<3+) urinary protein. Urinary albumin concentration was measured by using semi-quantitative dry immuno chemical screening strips (Micral II ^® ^test strips (Roche diagnostic GmbH Mannheim Germany). Micral Tests were performed on first morning urine collections and a value of more than 20 μg/min was judged as pathological. Chronic renal failure was defined by a serum creatinine level > 3 mg/dL [16].

Cardiovascular complications were ascertained using standard procedures. Coronary artery disease was identified by symptoms of definite angina pectoris or of definite past myocardial infarction and/or ECG changes consistent with previous myocardial infarction. Peripheral neuropathy was determined by Diabetic Neuropathy Symptoms (DNS) [17] and the Diabetic Neuropathy Examination (DNE) scores [18]. Neuropathy was considered to be present if DNS score was > 0 and/or the DNE score was >3. The presence of retinopathy was confirmed by fundus photography using digital camera.

### Statistical analysis

The data was coded and processed on IBM compatible computers, using the Statistical Package for Social Sciences (SPSS) software (version 14). Descriptive analysis, using standard statistical methods was performed. Chi-square tests or Fisher's exact test, independent t-tests and Pearson correlation coefficient were used to ascertain the association between MA and clinical outcome variables.

A multiple logistic regression model with backward selection (criterion: probability of *F *to remove ≥ 0.10) was used to estimate the effect of microalbuminuria (MA) among the sample population. The method enters all independent variables in the equation and then sequentially removes them. The variable with the smallest partial correlation with the dependent variable (*Y*_*i*_) is considered first for removal. If it meets the criterion for elimination, it is removed. After the first variable is removed, the variable with the smallest partial correlation remaining in the equation is considered next. The procedure stops when there are no variables in the equation that satisfy the removal criterion.

The variables included as predictors were: sex, type of DM, age group, duration of disease (in years), nationality and BMI group. That is in addition to a number of determinants of dichotomous (yes/no) outcomes related to DM including presence of coronary artery disease, diabetic retinopathy and neuropathy. Missing values for variables were replaced with the variable mean. ANOVA was used to estimate overall and individual significance of regression parameters. For overall significance the test statistic *F *was used to test parameters *B*_*ij *_for significance. For individual parameters the *t*-test was used. According to principles, the overall significance of the model was used as evidence for the suitability of the model.

## Results

### Socio-demographic characteristics of the study population

A total sample (n = 600) of male and female diabetic patients of all nationalities (UAE nationals and expatriates) resident in the Al-Ain district of Abu Dhabi emirate was selected; of whom, 513 agreed to enroll. Of the total sample, 52% were males, 27% aged 60 or above, 75% were UAE nationals and most patients (63%) were illiterate. Of the total sample 13% (95% CI: 11.0–14.6) were currently smoking while 8.2% (95%CI: 6.7–9.7) were ex-smokers and 76% were obese or overweight (Table 1).

**Table 1:** Baseline Characteristics of DM Patients in Al-Ain District, UAE, 2003–2004 (n = 513)

**Variable name**	**Prevalence of DM**	
	**n**	**Percent (95% CI)**

**Sex**		
Male	264	51.5 (47.2–55.8)
Female	249	48.5 (44.2–52.8)
**Level of Education**		
Illiterate	318	62.8 (58.6–67.0)
Completed primary school	99	19.6 (16.1–23.1)
Completed secondary school	60	11.9 (9.1–14.7)
Completed university	29	5.7 (3.7–7.7)
**Age group (Years)**		
40 or less	81	15.8 (12.6–19.0)
41 – 49	137	26.8 (23–30.6)
50 – 59	154	30.1 (26.1–34.1)
60 or above	140	27.3 (23.4–31.2)
**Nationality group**		
UAE	382	74.6 (70.8–78.4)
Other Gulf countries citizens	54	10.5 (7.8–13.2)
Arabs from other countries	54	10.5 (7.8–13.2)
Asians	22	4.3 (2.5–6.1)
**Smoking**		
Current smoker	64	12.8 (11.0–14.6)
Ex smoker	41	8.2 (6.7–9.7)
**BMI Group**		
Under weight (<18.5)	6	1.2 (0.2–2.2)
Healthy weight (18.5–24.99)	113	22.5 (18.8–26.2)
Overweight (25–29.99)	195	38.8 (34.5–43.1)
Obese (>30)	188	37.7(33.3–41.7)

### Clinical characteristics

The majority of the sample population (86%) had Type 2 DM; 49% were diagnosed incidentally and most of them (79%) had been diabetic for ≥10 years. Of the total population, 35% (95%CI: 30.8–39) had hypertension. The majority of patients (84%) were partially managed by oral hypoglycemic agents, 24% by insulin while two thirds were not following any exercise regime as part of their DM management. The analysis of glycemic control of patients using HbA_1C _showed that 38% (95%CI: 32.8–42.4)) had good glycemic control (Table 2). Dyslipidaemia, assessed by elevated total cholesterol, was present in 34% (95%CI: 30.0–38.8) and elevated triglycerides was present in 24% (95% CI: 19.9-27.9) of the sample population.

**Table 2:** Clinical Characteristics of DM Patients in Al Ain District, UAE, 2003–2004 (n = 513)

	n	Percent (95% CI)
**Type of DM**		
Type 1	68	13.6 (10.6–16.6)
Type 2	431	86.4 (83.4–89.4)
**Mode of Diagnosis**		
Incidental	245	48.5 (44.1–52.9)
Screening	39	7.7 (5.4–10.0)
Symptomatic	221	43.8 (39.5–48.1)
**Family History of DM**		
Present	270	54.3 (49.9–58.7)
**Duration of the Disease**		
< 1 year	33	6.6 (4.4–8.8)
1–5 years	199	40.0 (35.7–44.3)
6–10 years	161	32.3 (28.2–36.4)
11–20 years	98	19.7 (16.2–23.4)
>21 years	7	1.4 (0.4–2.4)
**Hypertension**		
Present	178	34.9 (30.0–38.8)
**Total Cholesterol**		
High (>5.60 mmol/L)	152	34.4 (30.0–38.8)
**Triglycerides**		
High (>2.10 mmol/L)	105	23.9 (19.9–27.9)
**HDL**		
Low (<0.91 mmol/L)	36	25.7 (18.5–32.9)
**LDL**		
High (>3.4 mmol/L)	70	53.4(44.9–61.9)
**Microalbuminuria**		
Present (>20 μg/min)	276	61.2 (56.7–65.7)
**HBA_1_c**		
Good control (<7%)	150	37.6(32.8–42.4)
Poor control (>7%)	246	62.4(57.6–67.2)

### Prevalence of nephropathy

Of the total sample, MA was assessed in 451 patients. The most common reason for missing cases was patients' failure to bring first void urine samples. The analysis of the sample population showed that 12.5% (95% CI: 8.1–14.1) had clinical proteinuria; 11.1% had trace, 1+ and 2+ and only 1.4% had gross proteinuria (3+ or greater). Chronic renal failure was found in 3 patients only. MA was present in 61.2% (95% CI: 56.7–65.7) of the sample population. The univariate analysis showed that MA was slightly more frequent in males (53.3% vs 46.7%) compared to females and was significantly higher among overweight and obese compared to the normal weight individuals (80% vs 18.8%) (p = 0.002). The presence of MA was statistically significantly associated with presence of chronic complications of DM such as coronary artery disease (p = 0.03), diabetic retinopathy (p = 0.07) and diabetic neuropathy (p = 0.01). MA was also more common among patients with type 2 DM compared to those with type 1, and the results were statistically significant (p < 0.04) (Table 3). The prevalence of MA showed no statistically significant associations with other covariates, including patient's age, nationality, disease duration, and presence of hypertension, dyslipidaemia or glycemic control.

**Table 3:** MA in Relation to Baseline Characteristics of the Study Population, in Al Ain District, UAE, 2003–2004 (n = 513)

**Variable name**	**MA Present**		**MA Absent**		
	**n**	**Percent**	**n**	**Percent**	**p-value**

**Sex**					
Female	129	46.7	96	54.9	0.09
Male	147	53.3	79	45.1	
**Type of DM**					
Type 1	42	15.8	16	9.2	0.04
Type 2	224	84.2	157	90.8	
**BMI**					
Under weight (<18.5)	1	0.4	4	2.4	0.002
Healthy weight (18.5–24.99)	51	18.8	43	25.4	
Overweight (25–29.99)	98	36.2	75	44.4	
Obese (>30)	121	44.6	47	27.8	
**Nationality**					
UAE nationals	209	75.7(70.6–80.8)	132	75.9(69.5–82.3)	0.09
Gulf countries citizens	25	9.1(5.7–12.5)	23	13.2(8.2–18.2)	
Arabs from other countries	34	12.3(8.4–16.2)	11	6.3(2.7–9.9)	
Asians	8	2.9(0.9–4.9)	8	4.6(1.5–7.7)	
**Coronary Artery Disease**					
Present	31	11.4	32	18.4	0.03
Absent	242	88.6	142	81.6	
**Neuropathy**					
Present	118	42.9	52	31	0.01
Absent	157	57.1	116	96	
**Retinopathy**					
Present	28	15.2	27	23.5	0.07
Absent	156	84.8	88	76.5	

A backward stepwise multiple logistic regression analysis with backward selection of factors that might independently be associated with development of microalbuminuria was performed on a number of predictors including, type of DM, age group, sex, DM duration (in years), nationality, BMI group, presence of coronary artery disease, diabetic retinopathy and diabetic neuropathy. The results demonstrated a statistically significant association between MA and the following covariates: male sex (adjusted OR: 0.48; 95% CI: 0.267–0.893), type 2 DM (adjusted OR: 4.309; 95% CI: 1.590–11.673), high BMI (adjusted OR: 0.614; 95% CI: 0.421–0.897), UAE nationality (adjusted OR: 0.706; 95% CI: 0.512–0.972), presence of diabetic neuropathy (adjusted OR: 5.811; 95% CI: 3.040–11.106) and retinopathy (adjusted OR: 2.800; 95% CI: 1.303–6.017) (Table 4). Patient age, disease duration, and presence of coronary artery disease were not significantly associated with MA and as such were removed by the model from the equation.

**Table 4:** Multivariate Analysis of Predictors for MA among DM Patients in Al-Ain District, UAE, 2003–2004 using stepwise Logistic Regression

**Variable**	**Regression coefficient**	**P value**	**Adjusted OR**	**95% CI**
Male gender	-0.717	0.02	0.488	0.267–0.893
Type 2 DM	1.461	0.004	4.309	1.590–11.673
UAE Nationality	-0.348	0.033	0.706	0.512–0.972
High BMI	-0.487	0.012	0.614	0.421–0.897
Presence of DR	1.030	0.008	2.800	1.303–6.017
Presence of DN	1.760	0.000	5.811	3.040–11.106

DR-Diabetic Retinopathy. DN-Diabetic Neuropathy

## Discussion

Diabetic nephropathy defined clinically as the presence of MA or overt nephropathy in patients with diabetes who lack indicators of other renal disease, is the most common cause of renal failure in the industrialized countries [4] and is now considered the commonest cause of ESRD worldwide. It is also widely acknowledged as an independent risk factor for cardiovascular disease [5]. MA is the first clinically detectable stage of involvement of the kidney, and affects between 20–40% of diabetic patients. Several epidemiological studies reported prevalence rates of MA ranging between 8–32% among type 2 diabetic Asian patients [19,20]. The variation in prevalence rates is most probably attributable to differences in diagnostic criteria, the stage of the disease, the method of assessment and ethnicity [21].

This study is the first cross sectional analysis assessing the prevalence of MA among diabetic patients in the UAE. The analysis showed an overall prevalence rate of MA amounting to 61% among the sample patients. This rate was clearly higher than the equivalent rates reported in the Arabian Gulf countries [22-24] and the differences could be attributed to the method of assessment or study design.

It is established that once MA is present, it is most likely to progress to proteinuria, over the next 5–10 years, in 20–50% of subjects. With the presence of MA, it is known that the decline in renal function varies but the average loss in glomerular filtration remains around 10–12 ml/min/year [25], and is accelerated by hypertension [26], though it is potentially reversible [27]. MA is also strongly associated with traditional cardiovascular risk factors and cardiovascular complications and events. Mogensen *et al *(1984) reported a significant increase in cardiovascular and total mortality in subjects with type 2 diabetes who had MA [28]. Dineen and Gerstein drew similar conclusions from a meta-analysis of 11 longitudinal studies [29]. MA is also associated with other diabetes complications such as retinopathy and neuropathy [30-33]. The association is held even after adjustment for other cardiovascular risk factors such as age, hypertension, smoking, dyslipidemia, abdominal obesity and hyperglycemia [34].

Similar to studies elsewhere, this study confirm that MA is statistically significantly associated with some adverse cardiovascular risk profile [35-38]. The univariate analysis showed that MA is significantly associated with the presence of coronary artery disease, diabetic retinopathy and neuropathy; though the latter was of borderline significance (p = 0.07). However, the multivariate logistic regression analysis demonstrated strong associations between MA and DM retinopathy (adjusted OR: 2.800; 95% CI: 1.303–6.017) and MA and DM neuropathy (adjusted OR: 5.811; 95% CI: 3.040–11.106).

Several risk factors have been identified for the presence of MA after adjustment for age and sex. Of significant importance were type 2 DM, UAE nationality, male sex, and obesity. As pointed out earlier, the results of many studies in other populations have shown that type 2 DM is commonly associated with higher prevalence of nephropathy [28]. Similarly, obesity, specifically with central fat distribution, is commonly known to be associated with urinary albumin excretion independent of blood pressure and plasma glucose [39-42].

Many studies have shown statistically significant associations between MA and insulin resistance [43] and the WHO definition of metabolic syndrome lists MA as one of the important components of the syndrome. Generally, our results were consistent with the findings of those studies [44,45]. However, our data showed no significant association between presence of MA and some known risk factors such as hypertension, degree of glycemic control, age and duration of diabetes. This could be attributed to the fact that our study was cross-sectional and as such its results cannot be used to correctly trace or establish the role for any of these factors in the development of MA in the sample patients.

The study has few limitations. Firstly, sampling from clinics may not be representative to all patients and/or the general population as undiagnosed subjects may be excluded. Secondly, the design was cross-sectional and therefore, causal relationships cannot be ascertained. Thirdly, it is known that DM is notoriously under-diagnosed, and therefore, the results shown might reflect only the tip of the iceberg. Fourthly, the proxy definition of DM was used in the study and auto antibodies screening such as Anti-GAD analyses was not assessed for all patients. As such it is likely that some type 2 diabetes are misclassified as type 1. Finally, the data from patients in Al-Ain may not represent all patients living in other parts of the UAE regions.

## Conclusion

In conclusion, the fact that MA could lead to adverse outcomes in patients, and the recognition that the risk factors for MA and its clinical course are amenable to intervention, provide a genuine case for action. MA is often incipient and can only be detected by special tests, such as the annual screening for MA, recommended by the American Diabetes Association, which is highly cost-effective. The availability of highly sensitive, cheap, dip-stick technology, strengthens the case for regular screening. Early detection of diabetic nephropathy, adoption of multifactorial interventions targeting its main risk factors and the use of renal-protective agents such as ACE inhibitors might reduce the progression of renal disease. Treatment of hypertension is a priority and the early attention to these measures will also ensure the reduction of cardiovascular mortality among diabetic patients.

## Competing interests

The author(s) declare that they have no competing interests.

## Authors' contributions

FA-M conceived the need for the survey in the UAE, participated in its design, and contributed to the interpretation of the results. ME-S, participated in the design of the study and the data analysis. OEN contributed to formulation of research question, manuscript reviews and data interpretation. FA-M and ME-S, OEN collaborated in writing up the manuscript. All authors read and approved the final manuscript.

## Pre-publication history

The pre-publication history for this paper can be accessed here:


